# The Benefits, Challenges and Impacts of Telehealth Student Clinical Placements for Accredited Health Programs During the COVID-19 Pandemic

**DOI:** 10.3389/fmed.2022.842685

**Published:** 2022-03-30

**Authors:** Rachel Bacon, Sian Hopkins, Jane Kellett, CaraJane Millar, Linda Smillie, Rebecca Sutherland

**Affiliations:** ^1^Faculty of Health, University of Canberra, Bruce, ACT, Australia; ^2^Speech Pathology, College of Health and Biomedicine, Victoria University, Footscray Park, VIC, Australia; ^3^Discipline of Speech Pathology, Faculty of Medicine and Health, University of Sydney, Camperdown, NSW, Australia

**Keywords:** telehealth, clinical placement, allied health, qualitative, COVID-19

## Abstract

**Introduction:**

Despite the advantages of telehealth, there has been a reluctance in its widespread adoption. During the COVID-19 pandemic, telehealth services and related placements increased internationally. Yet, there is currently limited research on the use of telehealth for student clinical placements.

**Aim:**

To explore the perceived benefits, challenges, and impacts of telehealth placements for key stakeholders (clients, students, clinical educators, and placement co-ordinators) in allied health courses.

**Methods:**

Stakeholder experiences with telehealth placements, undertaken within an Australian Allied Health University Clinic, were explored in virtual focus groups held between November 2020 and March 2021. These discussions used semi-structured interview questions, were audiotaped and transcribed verbatim. They were then thematically analyzed independently by two researchers, then cross-checked for consistency, using a qualitative descriptive approach, with reflexivity applied.

**Results:**

Twenty-six stakeholders from six allied health disciplines participated in seven homogeneous focus groups. Three themes were identified: (1) telehealth placements support competency development and graduate employability; (2) telehealth placements enable students to provide person centered-care; and (3) telehealth placements enabled innovation.

**Conclusion:**

Telehealth placements can make a valuable contribution as part of an overall placement program within accredited health courses and offer distinct advantages to student learning outcomes.

## Introduction

Telehealth is the remote delivery of health services using information and communication technologies to exchange health information. It can be both synchronous; two-way communication in real time (telephone and videoconference consultations) and asynchronous; one-way communication at any one time (text messaging and web-portals) (1). Evidence shows that telehealth can deliver high quality healthcare (2–5); transcends geographical, architectural, and physical distancing restrictions (6); and offers potential financial, efficiency, access, monitoring, and in-home advantages including patient empowerment and self-management (7). Telehealth's functionality, to bring together expert clinicians and carers (even if geographically dispersed), facilitates interprofessional collaboration which is known to improve clinical performance, patient outcomes, and patient satisfaction (8, 9). Despite these advantages there has been a reluctance in the widespread adoption of telehealth (10, 11).

During the COVID-19 pandemic, telehealth provided a means of delivering healthcare services while maintaining physical distancing and reducing the risk of viral transmission (12). Public funding for telehealth, [in Australia, this was through the Medicare Benefits Scheme (13)] was adopted in many countries, leading to an immediate increase in telehealth use (14, 15). The UK has seen a rapid expansion in video consultations. In Scotland, there was a 1,000% increase in video consultations during a 2-week period in March 2020 (16), albeit rising from a very small base. In Australia, the proportion of consultations provided by videoconference increased from 0.2% in February 2020 (prior to funding changes) to 35% provided by telephone and videoconference in April 2020 (17). The majority of these were provided by telephone (17), however, most allied health services also involved video consultations (18). The pandemic, however, also exposed telehealth knowledge and capacity gaps within the current health workforce (11, 14).

A significant challenge during the COVID-19 pandemic has been the provision of student placements (19). Most clinical training programs, such as degrees in speech pathology, nutrition and dietetics, occupational therapy and physiotherapy require students to undergo practical placements, under the supervision of a clinical educator. Traditionally, these are face to face placements in clinics, hospitals or schools, and both clinical educator and student are present with the client. COVID-19 restrictions, however, meant that such contact was not possible. There is some research supporting the use of telehealth placements within accredited health programs such as medicine (20–22), nursing (23–25), and allied health (26, 27). Telehealth can increase the placement capacity and scope (28), enable remote and retrospective supervision (27, 29), provide a safe learning environment for students during the pandemic (15, 20, 27), improve students' capability with telehealth improving employability (15), and offers opportunities for the development of interprofessional education (20, 27, 30–35). Signal et al. (7) argue that telehealth should become a core competency of practice, including in professionally accredited university courses (5).

This research responds to the current gap in accredited health placements (19) and the growth in telehealth technologies, funding, and services (10) resulting from COVID-19. While evidence shows telehealth can provide effective healthcare (2–5), research on telehealth for student clinical placements and interprofessional education is underexplored. The aim of this research was to explore the perceived benefits, challenges, and impacts of telehealth student clinical placements for key stakeholders.

## Materials and Methods

### Research Setting

This research was conducted within an urban Australian multidisciplinary allied health university clinic during the first “lockdown” of the COVID-19 pandemic. The clinic was established in 2010 and provides placements for 240 students annually from exercise physiology, dietetics, counseling, occupational therapy, optometry, physiotherapy, and speech pathology. In addition to discipline specific services, interprofessional services are provided through a cancer wellness clinic, Parkinson's clinic, and pediatric feeding clinic. Telehealth services were introduced in response to the pandemic from March 2020. All clients were situated in their own home during the sessions with students and clinical education staff generally collocated at the University clinic to provide the services via telehealth. In some cases, clinical educators and students were in separate locations, with students supervised remotely to provide telehealth. Clients used their own technology that was available to them, such as iPads, laptop computers and telephones. Other than the physical location of the client, student and clinical educator and the means of communication (telehealth, rather than face to face), the placements proceeded in a similar way to traditional placements provided by the clinic in terms of duration, client contact hours, student goal setting, and competency assessment.

### Procedure

This study was conducted between November 2020 and March 2021. It explored the experiences of a purposeful sample of key stakeholders (clients, students, clinical educators, and academic placement co-ordinators) directly involved in telehealth placements at the university clinic using focus groups. This method was chosen to make use of group dynamics to simulate discussion and to elucidate more complex issues (36). Focus groups were conducted in homogeneous stakeholder groups and, if required, multiple times were offered to maximize participation. Each focus group was attended by two researchers, one as a facilitator who was experienced in conducting focus groups and one as a scribe. The researchers facilitating the focus groups were directly involved with the clinic as university academics or clinicians, providing them with a richer understanding of the stakeholder roles, placement programs, and clinical services. Semi-structured questions informed by a placement quality framework (37) were developed by the first author in consultation with the research team. The questions for all participant groups covered overall experience, student preparedness, alignment with learning goals and competency development, benefits, challenges, and recommendations. Each focus group ran for 60-90 min and was conducted *via* Zoom Video Communications Inc (2020) (https://zoom.au/), which has been shown to be as effective as face-to-face focus groups when used by trained facilitators (38). All discussions were audiotaped and transcribed verbatim to maintain the integrity of the participants' responses.

### Data Analysis

Two researchers (RB; RS), who collectively had experience in qualitative research, clinical education, and telehealth, analyzed the transcripts using a qualitative descriptive approach (39) that was exploratory, inductive, and process orientated. Data were independently coded using descriptive labels, sorted into sub-categories and organized into themes, then crosschecked for consistency. The perspectives of the researchers as experienced clinical educators were acknowledged, and reflexivity applied in the coding process. Researchers met after the initial coding process to discuss and critique each other's interpretation; articulating their perspectives, identifying their assumptions, learning from each other's observations and ensuring that the emerging themes aligned with the research question. This research followed the criteria of the Consolidated Criteria for Reporting of Qualitative Research (COREQ) checklist for interviews and focus groups (40).

### Ethical Considerations

The University of Canberra Committee for Ethics in Human Research approved the present study (CEHR 4431) that confirms to the provision of the Declaration of Helsinki. All participation was voluntary and required written informed consent.

## Results

Twenty-six stakeholders participated in seven focus groups [clients (*n* = 3; *n* = 3), students (*n* = 5) clinical educators (*n* = 4; *n* =4) and placement co-ordinators (*n* = 2; *n* = 3)]. Table 1 provides more detail on the participants in each stakeholder group.

**Table 1:** Disciplines Represented Within Each Stakeholder Group.

**Stakeholder group**	**Health services/disciplines**
Students (Students enrolled in accredited tertiary health courses attending telehealth clinical placements at the University Clinic.) (*n* = 6)	Physiotherapy (*n* = 1) Exercise physiology (*n* = 2) Dietetics (*n* = 2) Speech pathology (*n* = 1)
Clinical Educators (Qualified clinicians responsible for both students learning and the delivery of the telehealth services at the University Clinic.) (*n* = 8)	Occupational therapy (*n* = 1) Physiotherapy (*n* = 2) Psychology(*n* = 1) Exercise physiotherapy (*n* = 3) Dietetics (*n* = 1)
Placement Co-ordinators (Academics responsible for the student clinical placement program mandated within accredited tertiary health courses with students attending telehealth placements at the University Clinic.) (*n* = 5)	Occupational therapy (*n* = 1) Speech pathology (*n* = 1) Physiotherapy (*n* = 1) Exercise physiology (*n* = 1) Dietetics (*n* = 1)
Clients (Community members receiving telehealth services from the University Clinic.) (*n* = 6) 2 clients attended more than one service	Occupational therapy (*n* = 2) Physiotherapy (*n* = 2) Psychology (*n* = 1) Exercise physiology (*n* = 1) Dietetics (*n* = 1)

Initial descriptions of the telehealth clinics were sought from all participants. As noted above, the transition to telehealth was the result of local COVID-19 lockdowns at the time. The move to telehealth was relatively sudden and all stakeholder groups commented on the rapidity of the transition to this new, and hitherto untried, service delivery model. For example, student “#S1-4” stated:

*I think it was something that no one was really prepared for, so like the supervisors themselves were learning at that time as well. Personally, I'd never heard of the word telehealth until it all came about, you hear on the news one day and then in placement the next day trying to do it and learn at the same time #S1-4*

Similarly, a clinical educator noted that there was a:

…*really fast rollout, it was a matter of, you know, days, maybe, maybe weeks to get it from not even having telehealth software to doing full telehealth video sessions #CE1-2*

While this was a common observation among the groups, a theme has not been developed around these ideas. Rather, this information is provided to understand the context of the telehealth clinics and the experiences of each of the stakeholder groups.

Three primary themes that emerged from the analysis of all stakeholder focus group discussions were that telehealth placements: (i) supported competency development and graduate employability; (ii) enabled students to provide person centered-care; and (iii) enabled innovation. Tables 2-4 provide subcategories and illustrative quotees as evidence for these themes.

**Table 2:** Theme 1: Telehealth placements support competency development and graduate employability.

**Subcategories**	**Illustrative quotees**
Competency development	**Student:** I think we still met our competencies on placement because we were still able to assess patients *via* video call. We obviously still had to adapt it, but it was still possible to do it and we were still able to implement evidence-based treatment. So, I think in terms of meeting competencies, it was good. S#1-1 **Clinical Educator:** In physiotherapy we have a national and standardized assessment tool that we use for physio students…so they get marked on nine different criteria and they are fairly broad criteria so I was surprised that you could sort of just translate that into telehealth #CE2-2 **Placement Co-ordinator**: There was not any issues with students being able to meet their competency standards, and I think that I was surprised that they actually did quite well transitioning to their hospital-based placements coming from, especially those who had done only telehealth, that they were not behind. And our students have finished and they have had their assessments moderated externally and there has not been any, any red flags raised from the students doing telehealth placements PC#1-2 **Client:** I was very impressed by the actual students. I thought they were able to establish a rapport with me very quickly, and they were able to convey a feeling that they were interested in how I was performing and that helped me to keep going. #C1-1
Communication advantage	**Student:** It really just strengthens your communication skills, and for me, it made me think before I spoke, and really pay attention to the power of the words I was using, and how I was phrasing things S#1-2 **Clinical Educator:** We mentioned the communication, that certainly has improved significantly. I think that is definitely a positive of the telehealth. I think the students were much stronger communicators, the students who were involved in telehealth. #CE1-1 **Placement Co-ordinator:** Their communication and risk assessment skills specifically were two areas where they really improved, and the communication is one that really, not that it surprised us, but it was amazing how much it improved, learning to describe things, yes, they could still use models and pictures, but if the patient has a very small screen, the model or the picture is not very clear. They had to overcome those kinds of barriers. PC#2-1 **Client:** …part of their learning I think and their own personal growth in trying to ask the appropriate questions that you ask in this distance mode as opposed as to you know, pretty obvious and familiar things that they'd be saying face-to-face when they could see what you were doing in the room with them. #C1-3
Employability	**Student:** I feel like I could be a lot more adaptable and flexible, and it is really good for keeping you up to date with using technology, being able to keep up to date, because it looks like it would be quite a good option for a lot of clients, even post-COVID. #S1-3 **Clinical Educator:** That little bit of an edge for the students when it comes to work readiness…while telehealth might not have been a feature of many dietetics workplaces, I know some like private practice dietetics practices who were quite specialized and have clients all over the country and overseas, and in fact, I've had one student who did a telehealth placement here who's gone on to do a placement at a practice like that and she was completely able to just jump straight in and know what they were doing with their telehealth. #CE2-3 **Placement Co-ordinator:** What we are seeing is that there are elements of telehealth continuing, and even with the federal government extending telehealth Medicare rebates and things like that we are going to see telehealth as a new normal. #PC1-1 **Client:** I think also it is important for the students to get the experience of telehealth and be all confident then when they are out in the big wide world that if necessary they can provide health assistance via telehealth if it becomes essential for them to do so. #C1-2

**Table 3:** Theme 2: Telehealth placements enables students to provide person centered-care.

**Subcategories**	**Illustrative quotees**
Continuation of care	**Student:** We set up a system to call each of the patients and make sure they had their exercise program, and then we set up a system in terms of checking in on them each week to know what they were doing, how they were doing it, whether they liked it, or if there needed to be any changes. #S1-1 **Clinical Educator:** The biggest, the best thing about it is that it allowed us to access people remotely when we could not actually see them in person, which was really important so that the clients still felt like they were getting supported and they were still able to progress with their treatment through us as well. #CE2-1 **Placement Co-ordinator:** A benefit was we could continue to deliver services in a way that we would not have been able to. #PC2-2 **Client:** …it was good to have that regular ongoing contact and follow up and interest in, how I was doing over, that period of time. I was delighted to be know, that I was known and know that I was being cared about, that was very important for me. #C1-2
Empowering	**Student:** I'd say less demanding on the client, because they didn't have to leave the comfort of their own home. S#1-2 **Clinical Educator:** We had to talk people through everything, including the assessment tests we would normally do to the client. I guess we had to coach them through a way of sort of assessing themselves to do that #CE1-2 **Placement Co-ordinator:** Because the child could be entertaining themselves in the room and I could actually communicate with both parents, which is more challenging in a clinic environment. For students, that was fantastic because largely the information we give for a pediatric speech clinic tends to be for mothers, but they had to learn how to adapt that information to the needs of fathers, or same sex partners, which they may not have had that experience had we not had telehealth. PC#2-2 **Client:** He could also do it from the comfort of his own home so that made him more likely to participate and more comfortable, knowing I could sit beside him and, and be with him during that, so I found it very helpful #C2-1
Accessible	**Student:** There was actually some clients from Sydney, from Dubbo, so we. Were able to target at a wider range of people. #S1-4 **Clinical Educator:** We can see people who are not able to actually transport to the actual clinic itself, so people who do not have a carer or they're finding it just too challenging getting out of their house because they don't feel well, we can still see how they move within their actual space and we can actually integrate an intervention within their current environment, so we find that it is actually working quite well. #CE2-4 **Placement Co-ordinator:** In the time of the pandemic, we had restrictions in terms of how many people could physically be in the clinic, and we had families that had five other siblings that had to come along, well we had rooms that could not let nine people in a room just for one-on-one therapy. So, to be able to do it *via* telehealth really, really helped. #PC2-2 **Client:** The travel time, yeah, really can add to it and, and not just the travel but yeah, organizing extra kids and things like that to, you know, who's going to look after them and that so that, yeah, the convenience of it, yeah. #C2-1

**Table 4:** Theme 3: Telehealth placements enabled new innovation.

**Subcategories**	**Illustrative quotees**
New ways of thinking	**Student:** It forces you to learn on the spot…the cues you would usually use in person probably are not going to work when you cannot assess a person when you're right there with them…it just forces you to think a bit “outside the box” about your language and communication you're using. #S1-5 **Clinical Educator:** This cohort will be more flexible and adaptable and they'll probably be open to trying new things and doing new things because they've seen it. Hopefully, they've got a broader concept of what the profession is and what the options are to them. #CE1-4 **Placement Co-ordinator:** It has got us thinking ‘outside the box', and actually the whole COVID has, and it has also potentially given us some ideas on expanding placements and potentially different placements in the future. So, from a curriculum point of view we are in a situation where we often have more students than we have placements, telehealth is giving us some ideas across the country as to how we can increase placements. #PC2-1 **Client:** If you said you didn't have a particular piece of equipment there was discussion around what could be the alternative, how it could be used and what you would need to do to make sure that you were getting the same effect that was intended. I mean, it was not an ideal world, but it actually ended up being pretty, pretty dam good. #C1-3
Changed practices	**Student:** We did a home assessment on somebody who was experiencing freezing, and it's really hard to explain, but basically in the clinic we can give them all the strategies we want, but if they do not practice it or try it at home, it won't make a difference. Being able to use a video, and they show us exactly where they're freezing, and then give them a strategy to use, I think that was a huge thing that we'd never even really considered before, and I think that's something they could even implement into their service. Even though everyone's come back to the clinic, if someone was struggling with that at home, that was something they could give as an assessment. I feel like it just opened up a new area that we could work in as well. # S1-1 **Clinical Educator:** One of the unexpected benefits was being able to use telehealth as a secure way for students to observe. I had a couple of occasions where I had students who were unable to come into the clinic, it was COVID related, and we were able to set them up on telehealth on a university device from home so they were able to perhaps be observing sessions so they weren't missing out. #CE2-3 **Placement Co-ordinator:** For us it was also focusing on “hands-off” treatment which is becoming better evidence-based. There's a lot of reliance on “hands-on” treatment, and patients are wanting “hands-on” treatment, whereas the evidence, especially when it comes to persistent pain, for example, is a lot more “hands-off,” and getting that message through. I mean we spend most of our curriculum teaching students “hands-on” stuff, and in the real world there's a lot of “hands-off” stuff. So, I think that was actually another good point that came up quite surprisingly. #PC2-1 **Client:** [The educator] occasionally wired me up for a week with her exercise tracker and that's a great motivator when you know that someone's going to sort of download your data after a week. #C1-2
Future visions	**Student:** I just completed an internship with some private practice dietitians, and just being able to observe the different types of clients that they're able to reach using telehealth which also extended to reaching clients overseas, in different time zones and all of that, to be able to educate people and connect with them, just over telehealth. #S1-2 **Clinical Educator:** I think we are going to produce graduates who are comfortable using telehealth and familiar with it, and that's changing views. It certainly changed my view about using telehealth and the accessibility of that and being willing to offer that. I'd be interested to see if that has good effects in the future for rolling out rural health and making services more accessible…in psychology especially we're thinking like, can we advertise further afield if we're offering telehealth to have more clients if we're offering a service rather than just in the local region. #CE1-3 **Placement Co-ordinator:** We shouldn't be willing to give up on these valuable experiences learnt during this time. We have to make sure that we keep these valuable skills, and use them even after the pandemic. PC#1-3 **Client:** Now that we know that telehealth is in existence, we know it works, even once people have been discharged from their clinics, to be able to have a telehealth from time to time, to ensure that even though you're now discharged you're staying on program, that there will be an ongoing connection and a concern about the things that are continuing to progress, that kind of seems to me as though now we have this platform that works well and that maybe a way to continue a service into the future. #C1-2

### Theme 1: Telehealth Placements Supported Competency Development and Graduate Employability

Three subthemes were developed in relation to this theme, namely *Competency Development*,

*Communication Advantage*, and *Employability* and are described below.

#### Competency Development

All stakeholders were clear that the telehealth placements enabled students to continue their placement through the COVID-19 pandemic and that they continued to develop a range of competencies related to their disciplines. Student competency continued to be assessed using discipline specific competency measures as they would have been had the placements been face-to-face. For many, being able to continue placements at all given the uncertainty of the times was of benefit. Further, telehealth enabled students to meet their placement learning goals and develop and demonstrate their competencies. However, some clinical educators expressed concern that the full range of skills and competencies could not be demonstrated *via* telehealth alone, particularly in fields where “hands on” skills such as assessments of strength or other body functions that are not possible to do remotely. Similarly, concerns were raised in disciplines where there was a need for students to learn to administer standardized assessments, and a clear need arose for access to assessment and intervention tools available and validated for use in telehealth. Challenges were also noted in the translation and transference of skills between telehealth and face-to-face tasks, with students required to transform their learning across contexts:

*They struggled to say, what can I take from this other context and put it into this new context? And you had to reassure them that actually a lot of the underlying principles are exactly the same, it's not like it is completely different. You've got to take that learning that you had before and implement it in this new setting, but I find that often some, often students about a lot of things can be quite black and white…The actual underlying principles are exactly the same, whether you're doing it on a screen or whether you're doing it face-to-face #CE2-4*

Further, while there is potential to develop in Interprofessional Collaborative Practice (IPCP) competency development this was not demonstrated in this study. In summary, while students were able to develop a range of competencies during their telehealth placements, given the challenges, there was a preference for telehealth to be part, rather than all, of a student's placement program.

#### Communication Advantage

A second subtheme was focused on the communication advantages that were apparent in the telehealth sessions, in terms of skill development for a range of stakeholders. Students and clinical educators noted that telehealth offered advantages over face-to-face placements in developing communication capabilities. This included telephone etiquette, being more explicit when giving and receiving feedback, coaching, written exercise prescriptions, and resource development. The provision of telehealth placements appeared to offer benefits to some students' learning. The nature of the interactions allowed educators to guide student's learning and students reported that the support, and being expected to focus on fewer elements at the same time, also scaffolded their learning.

*I think for our students, one of the things that the students commented was helpful was to do with scaffolding their learning…in the team-teaching model with one of educators, they would mute the session and could have a little bit of a chat and provide some support in a less intrusive way than they would do with a face-to-face consultation. And students actually found that really helpful, they felt like it helped them, particularly early on…their learning was scaffolded in the sense that starting with just the communication side of it meant they were focusing on that aspect of it without having to worry about the setup in the room and some of the other complexities when it became face-to-face. PC#1-2*

However, the need for strong communication skills, and reliance on verbal communication meant that telehealth was more challenging for others (e.g., students with English as their second language).

*I noticed that the student who was non-English speaking or second language English had a lot more difficulty with the telehealth. And I think it's also because they're often, you know you can often use gestures and non-verbal communication, not just to understand what the other person's saying but also to explain what you want to do*. #CE2-4

#### Employability

A third subtheme regarded the employability of students who had completed telehealth placements. Stakeholders indicated that not only did telehealth allow for the development of discipline specific competencies, it also helped to prepare students for the workforce, developing capabilities with telehealth and information technology, innovation and problem-solving skills and soft skills development (communication, flexibility, adaptability, reflective practice, risk-assessment).

*I think it really has set us up for the future…I think post-COVID people still will be implementing telehealth. I don't think that it's just a thing that we went through and we'll never do again…It helped with my communication, but then it also helped with assessing risks and things like that. And also, I guess because it was so fast moving, with quality improvement #S1-1*

### Theme 2: Telehealth Placements Enables Students to Provide Person Centered-Care

Three subthemes were developed regarding the provision of person-centered care: *Continuation of care, Empowering* and *Accessible Services*.

#### Continuation of Care

Despite the challenges of telehealth and the rapidity of moving to a new modality, students and clinical educators were able to continue to provide person-centered care. Clients in particular were clear that telehealth helped them to feel connected and cared for throughout the COVID-19 pandemic lockdowns.

…*knowing that they actually cared and they would follow through and you know, sort of having the sense of being part of a bigger whole but being a, being a separate person in that bigger whole and that they were paying special attention to me, I mean we weren't, we didn't meet in a group like this where everybody gave their feedback, it was just me and you know, the student and the lecturer in the background there as necessary. I value that continued interest in myself and my healing process. #C1-3*

Person-centered care, and the continuation of this, was also enabled by virtual home visits, with telehealth offering several “in home” advantages. The use of virtual home visits allowed the client to remain in the comfort of their own home, and enabled the student and clinical educator to see the client in their usual environment. Providing home-based sessions meant the client did not have to travel or organize child-care, increasing efficiencies. It also provided the health student with access to more accurate information such as food items and medication.

#### Empowering

Stakeholders reported that telehealth required clients to be more active participants in their health care delivery (doing *with* rather than *to*), for example:

*Students had to learn a lot more about coaching, parent coaching rather than necessarily just being the ones to be the therapists and do the direct intervention. #CE2-4*

For some clients, telehealth also meant playing a larger role in managing their own health and providing a pathway to greater motivation and accountability:

*We take pre-measures such as blood pressure and temperature and heart rate, but if we can teach people how to do that over telehealth then it actually allows us to provide them an extra way of support when we can't actually physically come to the clinic. So, I'd say that is something that we're going to keep offering. #CE2-4*

#### Accessible Services

Telehealth increased the scope, access and flexibility of the services offered by the clinic; a point noted by all stakeholder groups. Clients, particularly, could see the potential for telehealth to reach people in more isolated areas, and to provide more flexible services, including hybrid services. Telehealth in this study included telephone, videoconferencing sessions and monitoring services, allowing for accessibility across different modalities, according to the needs of the client, and the service being delivered. While all stakeholders identified benefits around accessibility, some noted that challenges continued to exist. The lockdown and technology limitations made it difficult to continue to deliver care to groups of clients using telehealth, and children had variable responses. The challenges of accessing technology were also highlighted by some stakeholders. Overall, while clients, students and clinicians were positive about the benefits of telehealth for providing person-centered care, there was preference for telehealth to continue as one mode of service delivery, rather than replace face-to-face services.

### Theme 3: Telehealth Placements Enabled Innovation

Three subthemes were developed in regard to how telehealth placements enabled innovation. These included: *New Ways of Thinking, Changed Practices and Future Visions*.

#### New Ways of Thinking

The rapid transition to telehealth had implication for all stakeholders. Students had to “think outside the box” as they transformed their learning from familiar face-to-face practices to telehealth. Educators and students now have the lived experience of changing to a new delivery mode broadening their concept of how their profession delivers healthcare. Placement co-ordinators were also inspired to explore new options to increase placement capacity. Clients also adapted to their circumstances and used innovative approaches to implement at home the care that was prescribed *via* telehealth.

#### Changed Practices

Changing to telehealth led to changes in other areas of practice. For example, the inclusion of other technologies such as exercise trackers, the increased use of “hands-off” treatments, new ways of assessing clients using video recordings, and interestingly, new educational approaches. The need for telehealth placements supported innovative supervision models including remote supervision and virtual observation, peer and retrospective supervision, along with an emphasis on community of practice and team approaches:

*Because we were learning at the same time it was real world and so the students really saw the vulnerability. I distinctly remember saying at times, I don't have the answers but I'm going to show you how we're going to work through this…It really went from a sense of, you know, I'm a clinical educator and you're the student, to much more of a team. #CE1-3*

For some services, competencies had to be assessed differently using telehealth, with innovation required to develop “hand-on” skills:

*We're not giving them ‘hands-on' experience, and for physio that's a major thing…we're needing to innovate how to augment telehealth-only placements with ‘hands-on' experience. For example, they might practice on each other in-between clients, so that they can actually learn how to do the ‘hands-on' technique…people would say we can't assess students if they're not seeing real patients because we can't see what they're doing, and you can still hear what they're thinking and hear them reason through how they're going to manage a patient. The problem of not being able to do ‘hands-on' stuff is something that we found a solution for. #PC2-1*

It was noted, however that while innovation was possible, and necessary, challenges remained. Changing supervision models in some disciplines meant a need for lower supervision student ratios, and as such, was reported to be more challenging for some supervisors.

#### Future Visions

Finally, telehealth gave the stakeholders a more positive perception of this mode of delivery and a desired to continue and expand its use. They saw telehealth as a way of overcoming geographical barriers, both in Australia and internationally, to allow students to access greater ranges of clients and competencies, and to develop broader relationships across a range of services.

## Discussion

Data analyzed from all stakeholders indicated that telehealth student placements were feasible, even when implemented as a rapid rollout in response to COVID-19. While challenges existed, the placements provided a range of benefits to key stakeholders. This study found that while there was a range of positive and negative experiences, telehealth placements provided continuity of placements and allowed students to learn and demonstrate a range of clinical competencies. In addition, clients received person-centered care that was meaningful and valuable to them, and telehealth placements fostered innovation in a range of areas.

A consistent theme through the data was that patients felt cared for and supported to continue their programs. Involvement in telehealth allowed them to maintain important connections to both the service providers, and to the groups that they had participated in prior to the pandemic. Similar findings have been reported in the literature. Holyk et al. found that telehealth users in rural and remote areas reported improved continuity of care and high levels of trust and satisfaction, particularly when telehealth services were part of a suite of local services (41). Other studies have found good levels of satisfaction with relationships with the healthcare professional and services delivered (42). As such, telehealth placements provide the opportunity to strengthen person-centered approaches in our health graduates.

This study found that using telehealth offered some advantages that empowered the client. Rather than having to go into an unfamiliar healthcare setting, people were able to stay in their own environment for their consultation. They could have their carers or family supports easily present with them. Childcare and travel arrangements were not required. For the students, this also enabled more accurate access to assessment data such as specific food items and medications. Telehealth offered the opportunity for efficient and affordable “virtual” home visits. This is similar to the findings of Record et al. telehealth also required the client to be a more active participant in their own healthcare (43). “Hands-on” interventions could not be undertaken *via* telehealth, rather the students were required to explicitly describe the intervention and do it “with” the client. This finding has been reported elsewhere in the literature (5, 44, 45); where a coaching approach was described with a greater emphasis on problem-solving, patient empowerment and education building capacity for self-management.

Telehealth was a relatively new concept for most of the stakeholders at the start of the transition away from face-to-face services. While telehealth has been in clinical use and in research for several decades (46), uptake prior to COVID-19 had remained modest, with telehealth largely considered a service for rural or remote clinicians and patients (47). Barriers including costs and technology as well as cultural factors have been documented (11, 40). With the advent of COVID-19 providing immediate need, and with technological advances providing new ways to overcome some barriers, there has been a renewed focus in research and practice on investigating the viability of telehealth for a range of populations (48, 49). Our research has shown similar findings. Expected barriers around technology, or lack of willingness to participate were able to be overcome. Telehealth services were therefore found to be viable not only for providing patient care, but also for the relatively novel task of providing student placements across several allied health disciplines, an important factor given the challenges of providing placements.

It is likely that addressing the role and practicalities of telehealth within the academic curriculum of clinical health training may be of benefit. Education about telehealth is not systematically included in all health degrees and for some students, particularly, this was a new area of practice that was entirely unfamiliar to them. While good experience and knowledge was picked up by these students during the course of their placements, it is likely to benefit all students to have improved knowledge of the suite of service delivery options that may be useful for a wide range of client groups. The challenges of including telehealth training in a systematic way across all health professions are significant (50), but given the rapid uptake of telehealth during COVID-19, it is imperative that such additions to curricula be considered.

Clinical placement shortages are well reported in the literature as a limiting factor for health workforce development and caps on accredited tertiary health courses (19, 20, 27). This research has exposed exciting opportunities to increase clinical placement capacity. In this study, telehealth provided a safe solution for clinical placement shortages that were a direct consequence of the COVID-19 pandemic. Using these placements helped to ensure on-time course completions for their students. While there is good evidence that telehealth can provide an equivalent healthcare service (2–5), this research suggests that telehealth can also provide appropriate clinical placement experiences that support competency development with unique advantages in some areas of employability skill development. Through this experience, placement convenors saw new opportunity to increase placement capacity and efficiencies including: (1) increasing the client-base of student-led health services no longer constrained by geographic limitations; (2) student placements opportunities in private and public telehealth services, with the option of attending placement virtually; (3) remoted supervision models; and (4) virtual support of supervisors and students. Based on our findings, however, a solution-focused approach is still required to overcome medio-legal, connectivity, and platform capacity barriers to make these new opportunities in clinical education innovation a reality.

In this research, clinical educators were positively challenged to reconsider their model of supervision. Innovative supervision models using telehealth, including co-supervision and virtual long-arm supervision, have been reported elsewhere (27, 51). The rapid-roll out of telehealth due to COVID-19 pandemic forced some supervisors to adopt a role more aligned to a team leader rather than student supervisor, changing the power-dynamic. This approach is consistent with Wenger's Community of Practice theory, where student learning is enhanced through a sense of belonging and participation within the healthcare team (52). This research also showed that in order to demonstrate competence with telehealth consultations, students had to transform their learning, transferring their knowledge and skills from the face-to-face context to telehealth. Such learning requires students to reflect on their experiences, recognize similarities and differences, seek new knowledge, and create new understandings (53), and supporting this was, at times, an important role for the clinical educators. This abstract analytical level of thinking is aligned with deep transformative learning (54, 55).

### Limitations

This study provides an in-depth qualitative exploration of a case example of a telehealth placement from the perspectives of key stakeholders. However, with a relatively small sample size and self-selected participants, these results cannot be generalized to all settings. Further, the study was conducted in the context of the COVID-19 pandemic and therefore, while reflexivity was used in interpreting this data, some conclusions drawn in this paper may relate more to this unique situation rather than more generically to telehealth. It is also important to note that some interviewers and participants may have been known to each other, with potential power imbalances as a result. Students, particularly, were assured that there were no right or wrong answers and that information about all experiences, positive or negative, were welcome and would be treated confidentially.

### Conclusions and Future Directions

Instigated by the COVID-19 pandemic, the rise in telehealth services has resulted in a new approach to clinical placement delivery. This study showed that telehealth could provide appropriate clinical placement experiences that supported competency development, modeled person-centered care, and offered unique advantages in some areas of employability skills development. It also exposed new opportunity to increased placement capacity and efficiencies. In particular, different approaches to student supervision were adopted that aligned with transformative educational practices. More research is required to optimize these potential benefits of telehealth placements.

Our research also provided us with insights into the needs of telehealth clinicians, students and clients both now and in the future. The importance of clear information for all stakeholders, such as tip sheets and guidelines, was clear, along with the development of innovative models of student supervision. Education, including the addition of telehealth specific education in clinical health training and support for clinical educators, is likely to have a long-term positive impact as telehealth becomes part of business as usual for all health care workers.

## Data Availability Statement

The raw data supporting the conclusions of this article will be made available by the authors, without undue reservation.

## Ethics Statement

The studies involving human participants were reviewed and approved by the University of Canberra Committee for Ethics in Human Research (CEHR 4431). The patients/participants provided their written informed consent to participate in this study.

## Author Contributions

RB, SH, and JK contributed to the research design and funding application. RB and JK completed the ethics application. RB, SH, JK, LS, and CM contributed to the data collection. RB and RS completed the data analysis and drafted the manuscript. SH and JK provided critical feedback and helped shape the research, analysis, and final manuscript. All authors contributed to the article and approved the submitted version.

## Funding

This research was funded by an Australian Collaborative Education Network (ACEN) Research Grant 2020.

## Conflict of Interest

The authors declare that the research was conducted in the absence of any commercial or financial relationships that could be construed as a potential conflict of interest.

## Publisher's Note

All claims expressed in this article are solely those of the authors and do not necessarily represent those of their affiliated organizations, or those of the publisher, the editors and the reviewers. Any product that may be evaluated in this article, or claim that may be made by its manufacturer, is not guaranteed or endorsed by the publisher.
